# An expressway traffic congestion measurement under the influence of service areas

**DOI:** 10.1371/journal.pone.0279966

**Published:** 2023-01-06

**Authors:** Lyuchao Liao, Zhengrong Li, Shukun Lai, Wenxia Jiang, Fumin Zou, Xiang Yu, Zhiyu Xu

**Affiliations:** 1 School of Transportation, Fujian University of Technology, Fuzhou, Fujian, China; 2 Fujian Provincial Expressway Information Technology Co., Ltd, Fuzhou, Fujian, China; 3 Fujian Provincial Key Laboratory of Automotive Electronics and Electric Drive, Fujian University of Technology, Fuzhou, Fujian, China; Southwest Jiaotong University, CHINA

## Abstract

Identifying traffic congestion accurately is crucial for improving the expressway service level. Because the distributions of microscopic traffic quantities are highly sensitive to slight changes, the traffic congestion measurement is affected by many factors. As an essential part of the expressway, service areas should be considered when measuring the traffic state. Although existing studies pay increasing attention to service areas, the impact caused by service areas is hard to measure for evaluating traffic congestion events. By merging ETC transaction datasets and service area entrance data, this work proposes a traffic congestion measurement with the influence of expressway service areas. In this model, the traffic congestion with the influence of service areas is corrected by three modules: 1) the pause rate prediction module; 2) the fitting module for the relationship between effect and pause rate; 3) the measurement module with correction terms. Extensive experiments were conducted on the real dataset of the Fujian Expressway, and the results show that the proposed method can be applied to measure the effect caused by service areas in the absence of service area entry data. The model can also provide references for other traffic indicator measurements under the effect of the service area.

## Introduction

Traffic congestion has become a growing global problem due to its impacts on economics, safety, and the environment [1]. Hence, identifying traffic congestion is critical to enhancing their operational efficiency and improving the level of service. Expressways have gotten increasing attention from academic and industrial circles as an essential element of traffic networks [2–5]. Specifically, service areas, as a critical part of the expressway, provide rest and vehicle parking services for travellers [6]. When users get into service areas, the travel time for users will be longer than before. This impact will decrease the accuracy of traffic congestion measurement. In addition, this impact negatively impacts estimating driving speed. Therefore, it is necessary to conduct a comprehensive analysis to quantify the impacts of service areas on expressway operations, verify their roles in measuring traffic congestion and propose efficient measures to erase their effects when measuring traffic congestion.

To measure traffic congestion more accurately, existing studies proposed different measurements based on the causes of congestion [7, 8] and the influences of congestion on operations [9] under different scenarios. These measurements can be divided into the following categories: 1) speed-based methods, such as speed reduction index [10, 11], speed performance index [12, 13]; 2) travel time-based method, for example, travel time reliability [13]; 3) delay-based methods, including delay rate [14], delay ratio [14]; 4) level of service, such as V/C [15].

Due to that the distributions of microscopic traffic quantities are highly sensitive to slight changes [16] in the real traffic system, the measurement of these traffic quantities faces abound of effects caused by different factors, such as work zone [17, 18], traffic incident [19–22], inclement weather [23–25], demand/capacity [26] and the environment around. Hence, the measurement of traffic quantities accurately arouses increasing interests from the academic and industrial circles. Existing studies could be generally classified into two categories: 1) analyzing the impact of dynamic traffic environment; 2) providing the adaptability of measurements. For example, Kwon, J summarized the impact of various traffic factors on the measurements’ effectiveness, including traffic-influencing events (traffic incidents, work zone activity, and weather), traffic demand (fluctuations in demand, special events), and physical features (traffic-control devices, inadequate base capacity) [27].

Furthermore, Kamga, C explored the temporal patterns of average travel time, standard deviation, and coefficient variation in urban areas under different weather conditions [28]. They found that inclement weather indeed increases average travel time. Caceres, H further estimated route travel time distribution by the probabilistic model with considering the time of day, inclement weather, and traffic incidents; then, he found that route travel time variability increases when the mean travel time increases [24]. Some studies show that when the length of a segment is long enough and the impact of traffic accidents is small, the value of Travel Time Reliability (TTR) will be normal [29].

However, as a part of expressway systems, service areas are always ignored when analyzing the impacts of factors on traffic congestion measurement performance. Most of existing studies focused on the pause rate [30–33], travel time estimation [34–36], and the service area itself [37–39]. Specifically, they often notice the impact of service areas while estimating travel time. For example, Y. Ohba noticed the unusual travel time and removed them [34]; unfortunately, he failed to distinguish between the data with long travel time caused by congestion and the data caused by service areas. Interestingly, YangYu employed large-scale streaming ETC transaction data to estimate fine-grained edge travel time, but he regarded these data of long travel time as the outliers and ignored them [36]. Additionally, the pause rate is proposed to evaluate as the rate of the number of vehicles entering the service area to the total vehicles during a specific time. Some works predict pause rate by judging whether the average speed meets the extremely low speed caused by service areas [32]. In fact, the influence of service areas is obvious and we can use the pause rate to figure out these impacts; however, the data of entering service areas are generally absent, making the pause rate hard to be obtained. Therefore, the accurate analysis of impacts caused by service areas is still a challenge for identifying traffic congestion.

In this work, we propose a traffic congestion measurement under the influence of expressway service areas with the ETC transaction data, named speed performance index correction method (SPIco). Unlike the PeMS dataset, the ETC transaction data show a finer granularity and provide a shorter length of segments. Firstly, we explore the travel time difference between the users who get into the service area and those who don’t. Then, the traffic congestion with the influence of service areas is evaluated by three modules: 1) the pause rate prediction module; 2) the fitting module for the relationship between effect and pause rate; 3) the measurement module with correction terms.

The contributions of this work could be summarized as follows.

- We firstly employ the Kolmogorov–Smirnov (KS) test to examine the travel time difference between users who get into the service area and others.
- We establish a linear model to examine the relationship between pause rates and measurement bias caused by service areas.
- We propose a framework SPIco to enhance congestion measurements for segments involving service areas in expressways under the absence of data in the service area.

The remainder of this work is organized as follows. Section 2 describes the research problem; Section 3 introduces the details of the SPIco; Section 4 describes the experiments on real ETC dataset, and the results are discussed in section 5. Finally, the conclusion is summarized in section 6.

## Problem definition

**Fig 1:**
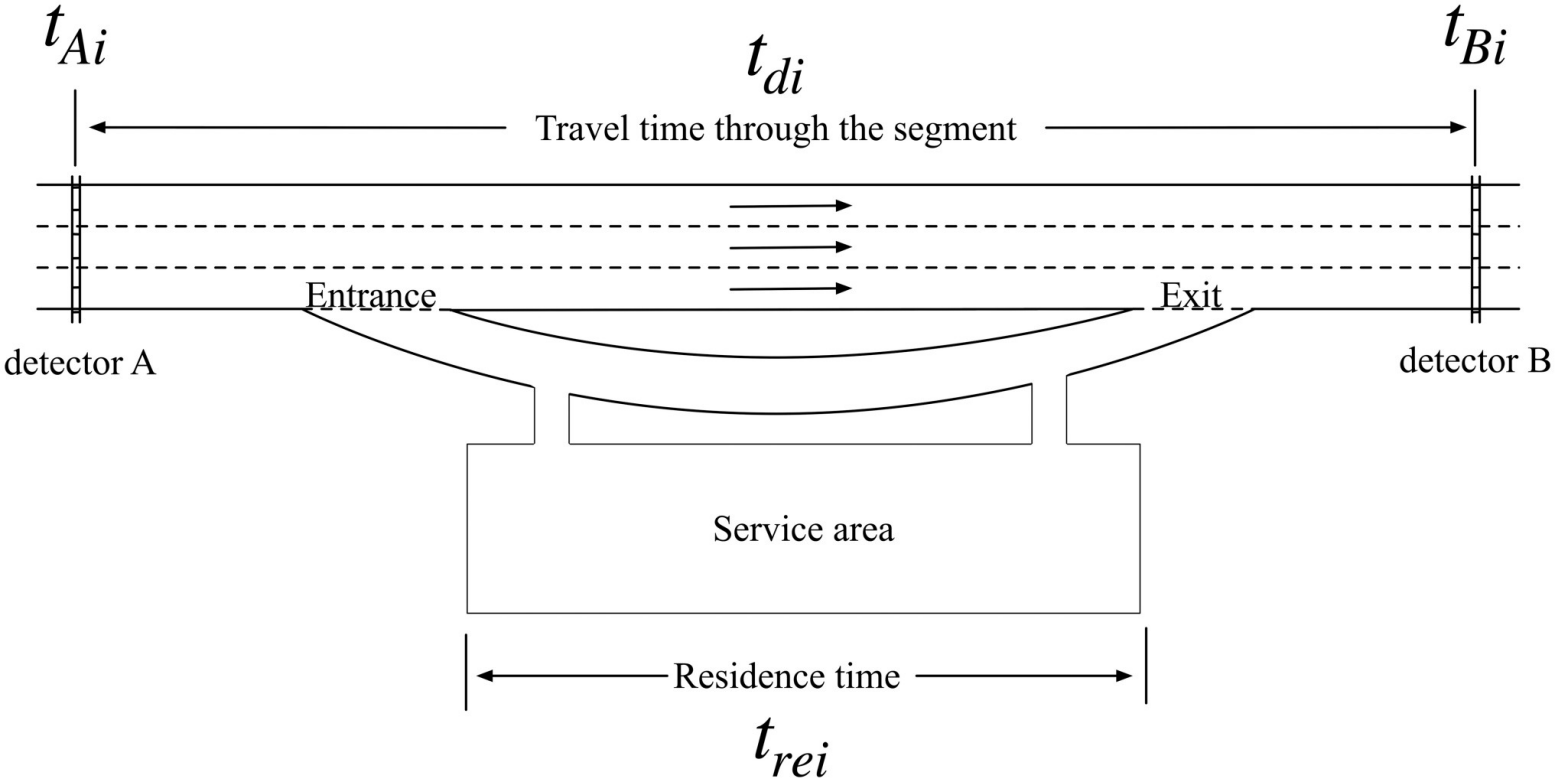
Sketch map of expressway service area.

To analyze the impact of service areas, we focus on an expressway segment with a service area in this work. As shown in Fig 1, detector A and B are road detectors (ETC gantries), and the service area are between two detectors. When drivers enter the service areas, their travel time will be longer, influencing the accuracy of traffic congestion measurement. Hence, we explore the relationship between pause rate and the influence to get the real travel time and erase the impact of service area.

When the *i*_*th*_ driver gets cross detectors, we can get the time *t*_*Ai*_ and *t*_*Bi*_, then we define the *i*_*th*_ driver’s travel time *t*_*ABi*_, and it can be calculated as Eq (1):

$$\begin{array}{c} t_{ABi}=t_{Bi}-t_{Ai} \end{array}$$ (1)

For the segment like Fig 1, *t*_*ABi*_ can be divided into two parts *t*_*di*_ and *t*_*rei*_. *t*_*d*_ *i* is the time of the *i*_*th*_ driver getting through this segment directly, and *t*_*rei*_ is the time of the *i*_*th*_ driver staying in the service area. Therefore, *t*_*ABi*_ could also be represented as Eq (2):

$$\begin{array}{c} t_{ABi}=t_{di}-t_{rei} \end{array}$$ (2)

The average travel time T_AB_ of this segment can be defined as Eq (3):

$$\begin{array}{c} t_{AB}=\frac{\sum_{i=1}^{m}t_{ABi}}{m} \end{array}$$ (3)

where *m* is the number of drivers. As shown in Eq (3), T_AB_ contains all drivers’ travel time. In addition, when *i*_*th*_ driver didn’t enter service areas, the value of *t*_*rei*_ will be 0 and *t*_*ABi*_ will be the same as *t*_*di*_, which is defined as the situation $t_{ABi}^{\prime }$. We could then get the average travel time of drivers who didn’t enter service areas by Eq (4):

$$\begin{array}{c} T_{\text{AB}}^{\prime }=\frac{\sum_{i=1}^{s}t_{ABi}^{\prime }}{s} \end{array}$$ (4)

Where *s* is the number of drivers who don’t enter service areas.

Without the data of $T_{\text{AB}}^{\prime }$, most existing works employ T_AB_ to measure traffic congestion, but $T_{\text{AB}}^{\prime }$ is better to reflect the real state of the segment. Unfortunately, it’s hard to get $T_{\text{AB}}^{\prime }$ directly. In this work, we aim to estimate the real state by analyzing the relationship between T_AB_ and $T_{\text{AB}}^{\prime }$. At first, we define this subtractive value between them as ΔTAB, as shown in Eq (5):

$$\begin{array}{c} T_{\text{AB}}^{\prime }=T_{\text{AB}}-\Delta T_{\text{AB}} \end{array}$$ (5)

In this work, we explore the impact of service areas to ΔT_AB_ by mapping ΔT_AB_ to the *bias* of SPI and exploring the relationship between bias and pause rate. The pause rate can be calculated as Eq (6) [30]:

$$\begin{array}{c} \text{rate}=\frac{m-s}{m} \end{array}$$ (6)

where *m* represents the number of all users in the statistical time and *s* represents the number of users who don’t enter service areas in the statistics time.

## Methods

In this section, after judging whether the service area affects the measurement of traffic state, we propose a speed performance index correction method, named SPIco. At first, we employ ETC transaction data and entrance data of service area to extract and predict pause rate by XGBoost, and then we explore the relationship between bias and pause rate. Finally, we generate a correction term SPIco for estimating and correcting the impact from service areas.

**Fig 2:**
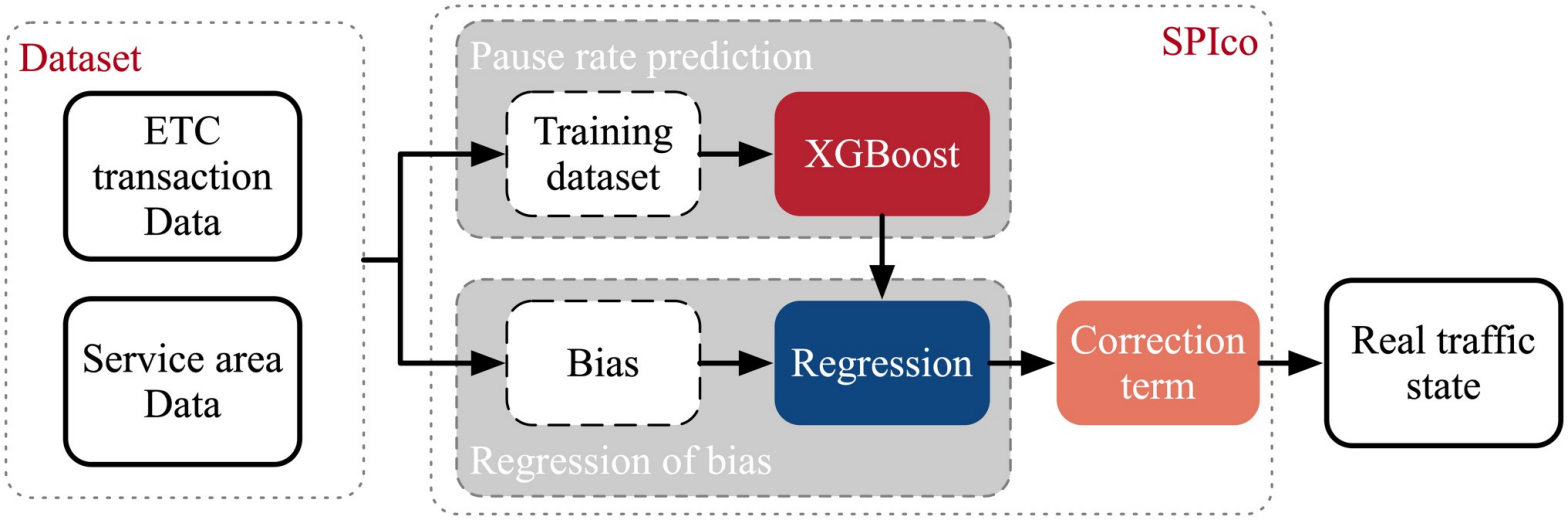
Proposed research framework.

As shown in Fig 2, SPIco contains three modules: 1) Pause rate prediction; 2) Regression of bias; 3) Correction term. Firstly, we extract the training dataset and the bias from ETC transaction data and service area data. Then in the module of Pause rate prediction, the training dataset is put into the XGBoost model to predict the pause rate. After predicting the pause rate, we explore the regression relationship between bias and pause rate. Then we obtain the corresponding bias with the predicted pause rate and put them into the correction term module. At last, the real traffic state is put out with the correct term.

To make sure SPIco still work under absence of data for service areas, we use ETC transaction data and entrance data of service area to extract pause rate and the correspond variables as input of XGBoost. Then we can predict the pause rate under absence of data for service areas. To fit the relationship between bias and pause rate, we calculate bias and use linear regression to fit the curve. At last, we add the correction term into SPIco. Fig 2 presents the proposed research framework.

### Significance test of difference

The significance test of difference (STD) is used to examine whether there is a difference between two samples and whether the difference is significant in scientific experiments. To figure out whether there is a difference in the distribution of travel time between users who get into the service area and users who don’t, the steps of the difference test are as follows:

- Proposing a hypothesis *H*_0_. In this work, *H*_0_ is that there is no difference in the distribution of travel time between different user groups.
- Using Kolmogorov–Smirnov (KS) test to examine the truth of the null hypothesis.
- Compare it to a reference value to establish significance, the P-value. Based on that, either reject or not reject the null hypothesis (*H*_0_).

KS-test is a nonparametric test of the equality of continuous (or discontinuous), one-dimensional probability distributions that can be used to compare two samples [40]. It quantifies the distance between the empirical distribution functions of two samples.

### Speed performance index

Speed performance index (SPI) is a measurement of traffic congestion, and it can be defined by the ratio between vehicle speed and the maximum permissible speed, as shown in Eq (7) [12]:

$$\begin{array}{c} \text{SPI}=(\frac{v_{avg}}{v_{max}})\times 100 \end{array}$$ (7)

where *v*_*avg*_ is the average speed of the vehicles within the segment or road; *v*_*max*_ is the maximum permissible speed of the road segment. Previous studies define the value of maximum permissible speed differently, including road-designed speed and the average vehicle speed during midnight40 (24:00–1:00) [41]. In this work, we use road-designed speed to simulate the actual traffic condition more accurately.

From Eq (7), it is evident that the value of SPI ranges from 0 to 100. Previous studies present that the value can be classified with three quantiles, and the classification criterion of the road traffic state is shown in Table 1.

**Table 1:** Speed performance index with traffic state.

SPI	Traffic state level	Traffic state description
[0, 25]	Heavy congestion	Low average speed, poor road traffic state
(25, 50]	Mild congestion	Lower average speed, road traffic state is weak
(50, 75]	Smooth	Higher the average speed, road traffic state is better
(75, 100]	Very smooth	High average speed, road traffic state is good

### SPIco to correct traffic congestion measurement with expressway service areas

#### Pause rate prediction

In this work, we classified features into three parts: temporal features, traffic flow features, and traffic composition features.

1. Temporal features
  The temporal-based feature contains the periods of a day (15-minutes for each time interval) and weekday indicator (if the day is a workday, the value of this feature will be 1, otherwise 0). The temporal-based features are as follows:
  $$\begin{array}{c} \gamma ={\left(\gamma_{1},\gamma_{2}\right)}^{T} \end{array}$$ (8)
  where *γ*_1_ is the period, and its value ranges from 0 to 95; *γ*_2_ is a binary whose value is set to 0 for a workday and 1 for a non-workday.
2. Traffic flow feature
  More users are willing to get into service areas when the traffic flow is large and the traffic condition is heavy. Therefore, the traffic flow has also been considered, and the feature is shown as follows:
  $$\begin{array}{c} v=(v_{1},v_{2},v_{3}\ldots \;v_{n}) \end{array}$$ (9)
  Where *v*_*i*_ represents *i*_*th*_ traffic flow.
3. Traffic composition features
  Given that drivers who use different vehicle types have different travel behaviors. For example, if the vehicle is an electric vehicle, the possibility of the user getting into service areas is higher than that who use traditional vehicles. Also, coaches which carry more people are more likely to get service than private cars. Therefore, the traffic composition feature is as follows:
  $$\begin{array}{c} \theta ={\left(\theta_{1},\theta_{2},\theta_{3},\theta_{4}\right)}^{T} \end{array}$$ (10)
  where *θ*_1_, *θ*_2_, *θ*_3_ and *θ*_4_ represent the percentage of vehicle groups with different vehicle types. *θ*_1_ represents the blue color which refers to fuel cars; *θ*_2_ represents the yellow color which refers to large vehicles such as trucks or buses with more than 2 tons (including trucks with ten seats or more), agricultural vehicles, tricycles, trailers, coach cars, and operational vehicles, such as taxis and buses; *θ*_3_ represents the green color which refers to new-energy vehicles, and *θ*_4_ represents the other color of the vehicle plate.

**Fig 3:**
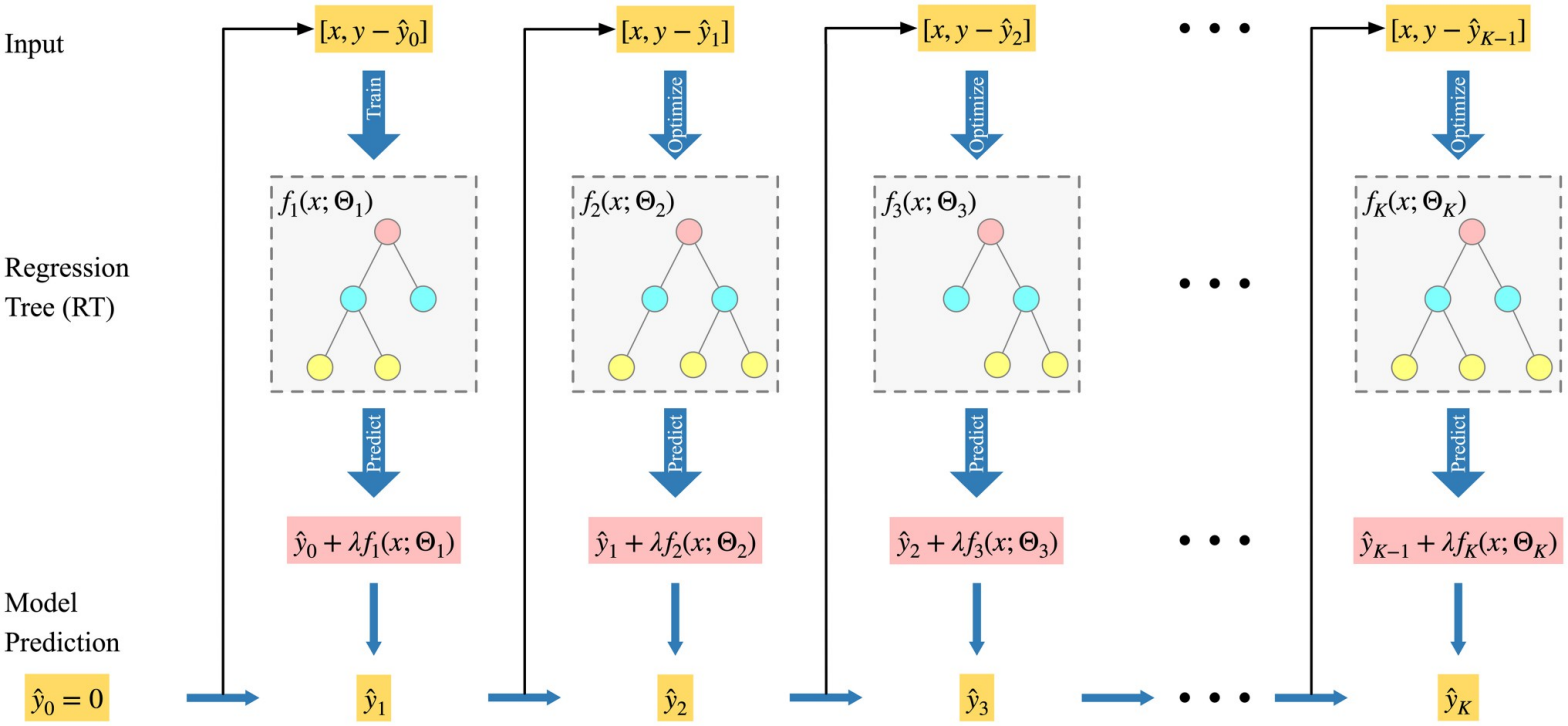
XGBoost schematic.

XGBoost is an integrated learning method based on the boosting algorithm [42], which uses multiple base learners to reduce the model error. Each base learner is relatively simple to avoid over-fitting. The schematic of the model is shown in Fig 3. In this model, the next learner’s generation depends on the previous learner’s results.

We extract the feature vector from raw ETC transaction data and the data collected by the entrance of the service area in the expressway. After labeling the data, we set the dataset as *S* = {(*x*, *y*)}, where *x* = (*x*_1_, *x*_2_, *x*_3_, …, *x*_*n*_)^*T*^, and *x*_*i*_ = (*γ*_1_, *γ*_2_, *v*_*i*_, *θ*_1_, *θ*_2_, *θ*_3_, *θ*_4_)^*T*^ (*i* = 1, 2, …, *N*) which represents the feature vector of the *i*_*th*_ sample. *Y* = (*y*_1_, *y*_2_, *y*_3_, …, *y*_*n*_)^*T*^(*i* = 1, 2, …, *N*) represent the pause rate corresponding to *x*_*i*_. The XGBoost model has K decision trees, and the prediction result is shown in Eq (11) [42]:

$$\begin{array}{c} {\hat{y}}_{i}=\sum_{k=1}^{K}f_{k}(x_{i}),f_{k}\in F \end{array}$$ (11)

where *K* represents the number of trees, *f*_*k*_(*x*_*i*_) represents the predicted value of the *k*_*th*_ decision tree on sample *x*_*i*_, and *F* represents the integrated classifier composed of all decision trees.

XGBoost does not directly minimize the above loss function as the training target but adds the complexity of the tree to the above formula to avoid overfitting. Therefore, the objective function consists of the loss function and the regularization item, as shown in Eq (12):

$$\begin{array}{c} obj=\sum_{i=1}^{n}L(y_{i},{\hat{y}}_{i})+\sum_{k}^{K}\Omega (f_{k}) \end{array}$$ (12)

where $L(y_{I},{\hat{y}}_{I})$ is the loss function, and then we use Mean Square Error (MSE) for regression, as shown in Eq (13):

$$\begin{array}{c} L(y_{i},{\hat{y}}_{i})=\frac{{\left({\hat{y}}_{i}-y_{i}\right)}^{2}}{n} \end{array}$$ (13)

Ω(*f*_*t*_) is L1 regularization, as shown in Eq (14):

$$\begin{array}{c} \Omega (f_{k})=\alpha T_{k}+\frac{1}{2}\alpha {∥W_{k}1∥}_{1} \end{array}$$ (14)

where *α* represents the regularization penalty coefficient, which takes values in the range of [0, 1]. *T*_*k*_ presents the number of leaves of the *k*_*th*_ tree and *W*_*k*_ represents the leaf weight of the *k*_*th*_ tree.

#### Regression of the bias

To obtain the bias, we divide the original data into two typical samples (pure dataset, original dataset). Pure dataset indicates that do not contain the data that vehicles entering service areas, and the original dataset is the whole sample. We then obtain the bias by calculating the difference of SPI under the two samples.

$$\begin{array}{c} bias={\text{SPI}}_{pure}-{\text{SPI}}_{original} \end{array}$$ (15)

where SPI_*original*_ represents the value of SPI under effect by service areas, and SPI_*pure*_ is the value of SPI without being affected.

In this work, we explore the relationship between bias and pause rate by linear regression, whose function is as follows:

$$\begin{array}{c} \hat{bias}=\omega •rate+b \end{array}$$ (16)

where *rate* is the independent variable pause rate, *ω* and *b* are parameters of the linear model, and the $\hat{bias}$ is the regression value calculated by a linear model.

#### Correction term

After prediction and regression, we established the measurement named SPIco to measure the traffic congestion under the effect of the service area. When the road segment contains a service area, the value of SPI will be lower. Therefore, when we use SPI_*pure*_ − SPI_*original*_ to get the bias, the value of the bias will be positive. Then, we add a correction term to make the value more accurate for obtaining the true value of SPI. The correction term is shown in Eq (17):

$$\begin{array}{c} correction=\hat{bias}\left(\hat{y}\right) \end{array}$$ (17)

where the $\hat{bias}$ is the regression value, and the $\hat{y}$ is the prediction value of the pause rate.

Finally, the novel measurement method, named SPIco, is shown in Eq (18):

$$\begin{array}{c} \text{SPIco}=(\frac{v_{avg}}{v_{max}})\times 100+correction \end{array}$$ (18)

### Evaluation metrics

In this work, we employed RMSE and MAE to evaluate the measurement performance of SPIco [43].

$$\begin{array}{c} RMSE(X,h)=\sqrt{\frac{1}{n}\sum_{i=1}^{n}{\left(h(x_{i})-y_{i}\right)}^{2}} \end{array}$$ (19)

$$\begin{array}{c} MAE(X,h)=\frac{1}{n}\sum_{i=1}^{n}|h(x_{i})-y_{i}| \end{array}$$ (20)

Where *h*(*x*_*i*_) is the vector of observed values, *y*_*i*_ is the vector of predicted values, and *n* is the number of samples.

## Data

**Fig 4:**
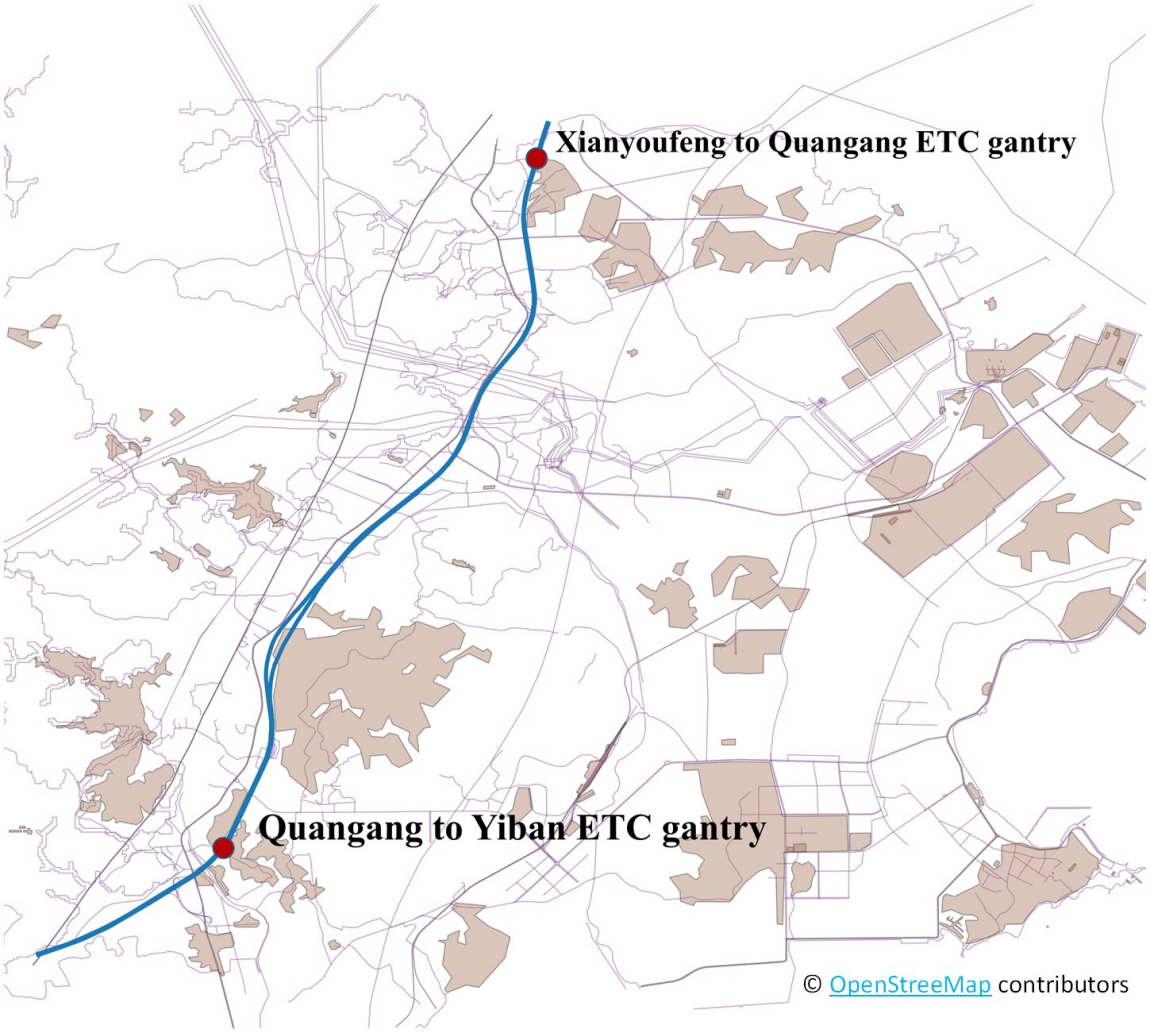
Illustration of the case study corridor.

Firstly, a case study was conducted to verify the difference between the two scenes and the adaptability of the proposed measurement. Specifically, we select a road segment on Shenhai Expressway in the Quangang District, Quanzhou City, Fujian Province, China, to analyze the difference and measure the traffic state. As shown in Fig 4, this segment is between two ETC gantries (from Quangang to Yiban and from Xianyoufeng to Quangang, in up and down direction); the length of this segment is 15.695 km. The raw data is collected by the ETC system when the vehicle crosses the gantry, like passenger ticketing records in a metro system. The raw data contain the time collected, the time to enter the expressway, the entrance name, and the vehicle license plate. For the service area, the data collected by video data, we extract the main features containing the entrance of a service area, the time collected, and the vehicle license plate. All the data features are shown in Table 2.

**Table 2:** Variables of raw data.

Variable	Description
ETC transaction data	
VEHPLATE	The vehicle license plate
TRADETIME	The time of collected
NODENAME	The name of entrance
ENTIME	The time of enter expressway
Service area data	
Area	The location of vehicles enter service area
VEHPLATE	The vehicle license plate
TRADETIME	The time of collected

## Results

### Descriptive statistics analysis

**Fig 5:**
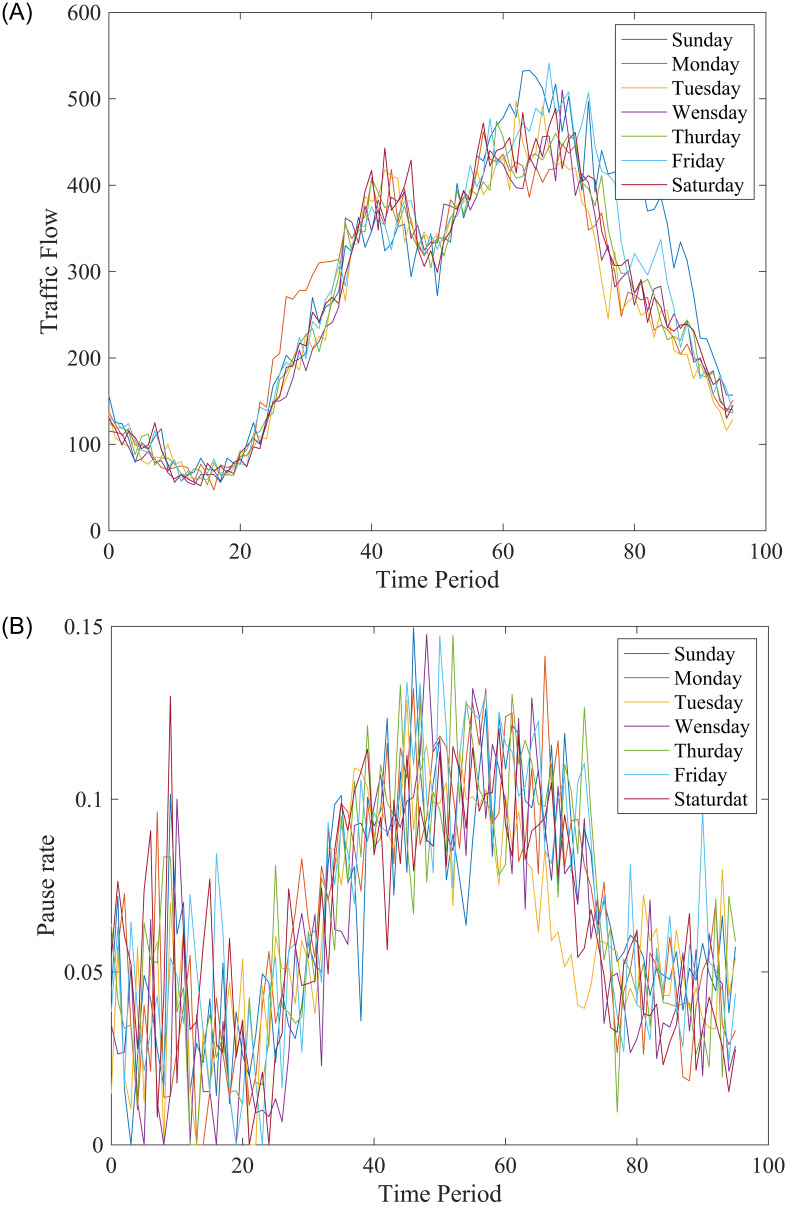
Time dependence of traffic flow and pause rate change curve. A: Time dependence of traffic flow. B: Time dependence of pause rate.

After briefly describing the data, we tested the difference in travel time between users who get into the service area and those who don’t get into the service at first. Then, we plot the traffic flow change at different periods by continuous week data, as shown in Fig 5(A). To figure out the relevance of the pause rate, time period, and the feature workday, we plot the pause rate under the change of time period. As shown in Fig 5(B), the pause rate is positively correlated with traffic flow, and we can also find that the pause rate is less affected by the feature workday and is more volatile.

**Fig 6:**
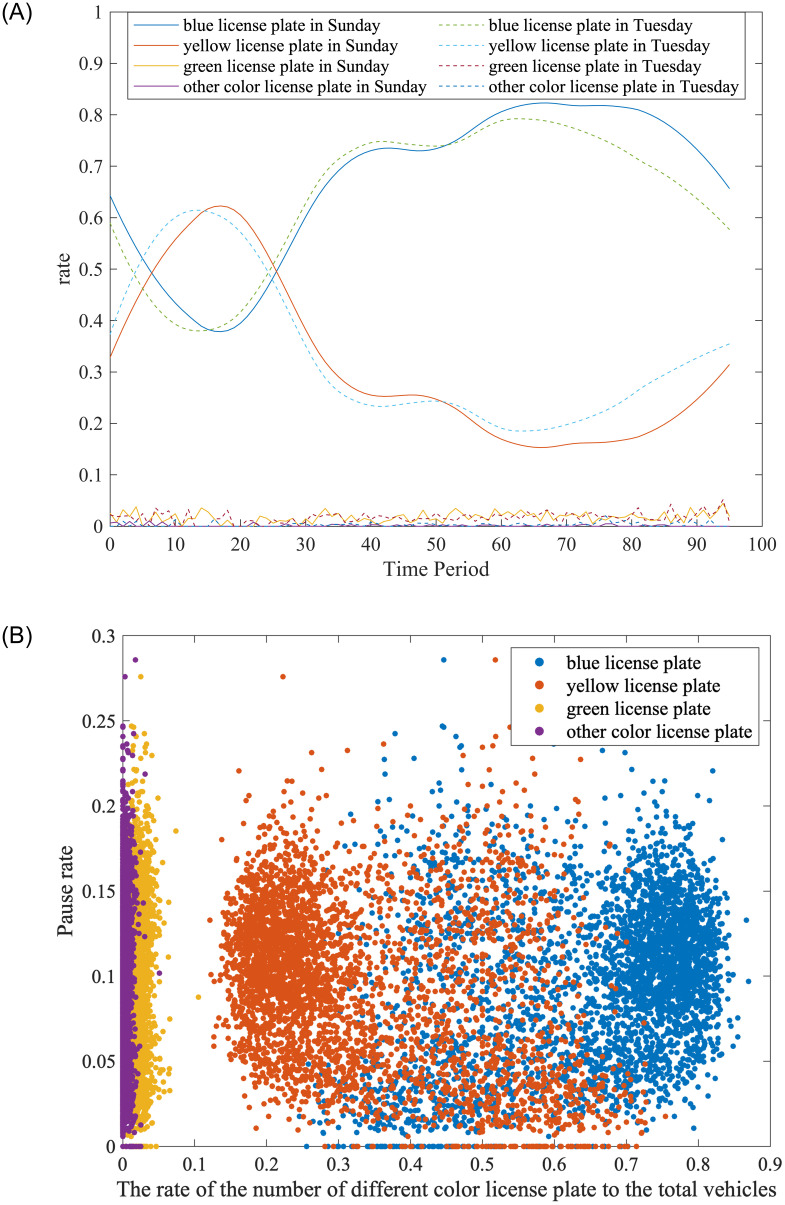
Correlation of traffic composition with time and pause rate. A: Time dependence of traffic composition. B: Correlation of traffic composition with pause rate.

To explore the relationship between traffic composition and pause rate, we plot the change of traffic composition and the scatter of pause rate, shown in Fig 6. Fig 6(A) shows the change of traffic composition in the workday and non-workday. The x-axis is the time period, and the y-axis is the rate of the number of different vehicle types. The solid lines represent the rate of different vehicle types under non-workday, and the dashed lines represent the rate under workday. From 2:00 to 5:00, the number of yellow license plate vehicles is more than that of blue. In comparison, the number of blue license plate vehicles is more than the yellow from 6:00 to 24:00. This is because the yellow license plate vehicles are mainly trucks and coaches, the trunks tend to travel from 2:00 to 5:00, and the blue license plate vehicles are mainly the private fuel cars. In general, the rate of green license plate vehicles and other colors is low.

As shown in Fig 6(B), the purple and yellow represent the green license plate vehicles and other color license plate vehicles; the blue and red represent the yellow license plate vehicles. The x-axis is the rate of traffic composition, and the y-axis is the pause rate. As shown in Fig 6, the blue license plate vehicles are positive to the pause rate; the yellow license plate vehicles are negative to the pause rate; the green license plate vehicles and the other color license plate vehicles have less correlation with the pause rate. The descriptive statistics of these variables are presented in Table 3.

**Table 3:** Variable description.

	Variables	Mean	St.d.	Min.	Max.
Flow feature	Flow	251.62	124.73	42	735
Time features	Time Period	47.5	27.71	0	95
	Workday	0.68	0.46	0	1
Traffic composition features	Blue license plate rate	0.65	0.14	0.14	0.87
	Yellow license plate rate	0.68	0.46	0	1
	Green license plate rate	0.018	0.01	0	0.11
	Other color license plate rate	0.003	0.004	0	0.051

### Difference test

**Fig 7:**
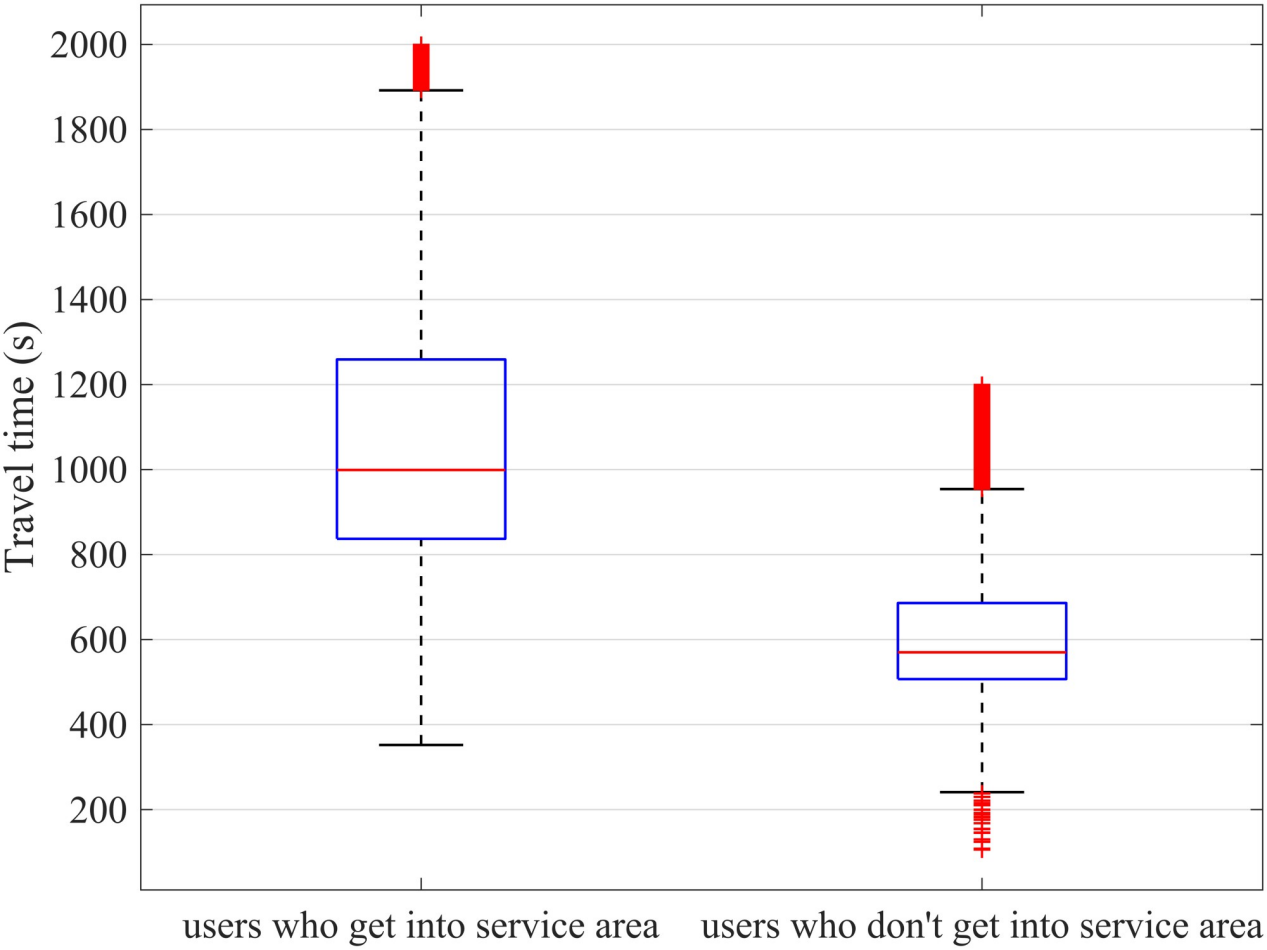
Boxplot of travel time.

We select a segment and use a boxplot to plot the travel time from 13, May 2021 to 31, May 2021 to analyze the difference between the two samples. Fig 7 shows the boxplot of travel time for two scenes, the left item is the travel time for users who enter the service area during the travel, and the right item is the opposite. Because of the personalized service of service areas, such as lunch, taking a rest, and other activities, the travel time for users who get into the service area is much larger than those who go through the road directly in Fig 7. Hence, they need more time to finish the travel; it can be seen in Fig 7 that the upper boundary of the right boxplot lies in the lower 50% of the left boxplot. Generally, the spread and the range between the two scenes are very different, and the data for users who don’t get into the service area are more concentrated.

**Fig 8:**
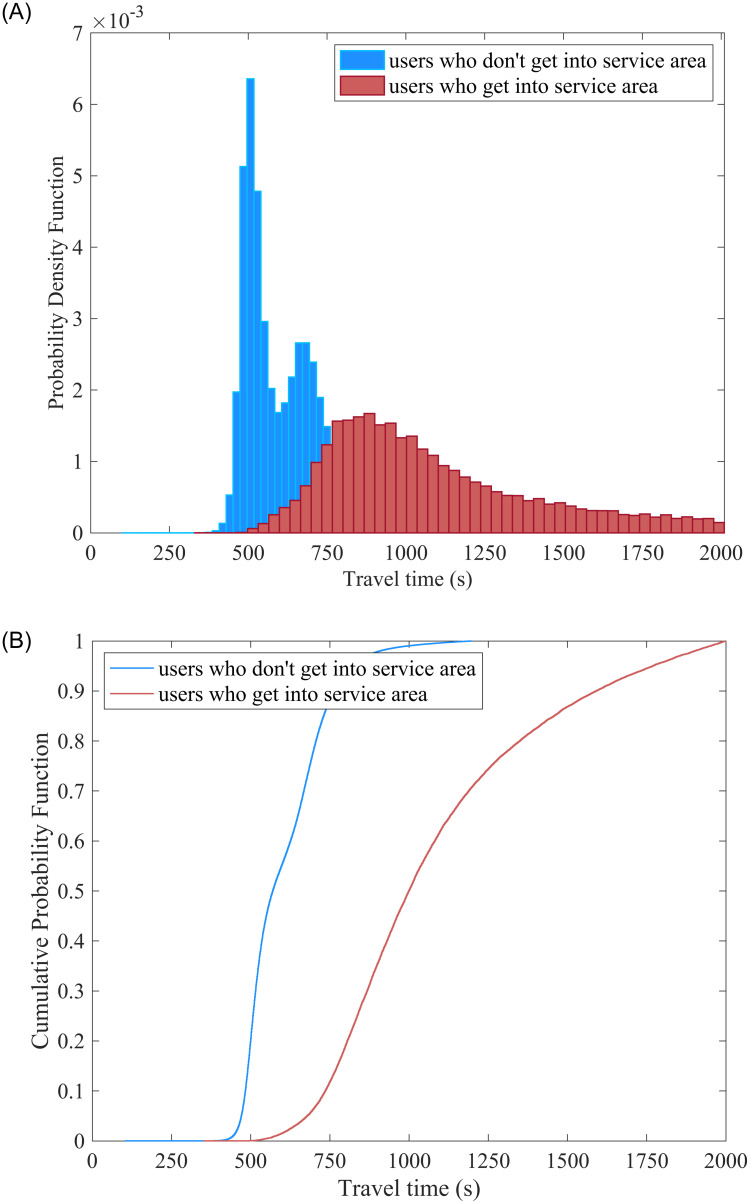
PDF curves and CDF curves. A: PDF of travel time. B: CDF curves of travel time.

To explain the distribution of the two scenes more intuitively, we compared the travel time distributions in Fig 8. It is obvious that the distributions of the two scenes are different. The travel time distribution for users who get into service areas is more dispersed than others; this distribution is unimodal and skewed. But for travel time, when users do not get into the service area, the distribution is bimodal. Specifically, one of the modal centers is around 500s, and another is around 700s. We can transform time into speed, the first is about 113 km/h, and the second is about 80 km/h. Referring to Chen’s study, he compares the travel time under the free flow and peak period conditions, and the result shows that the distribution for these two conditions is different [41]. Therefore, in this case study, the first modal is the free flow travel condition, and the second is the peak period condition.

After briefly viewing travel time distribution, we use the KS test to conduct a hypothesis test to verify the difference between these two conditions. In this study, the null hypothesis *H*_0_ is that there is no difference between the travel time under the two scenes, and the alternative hypothesis *H*_1_ is that there is a difference between the travel time under the two scenes. The result is rejecting the null hypothesis *H*_0_ and receiving the alternative hypothesis *H*_1_ that there is a difference between the travel time under the two scenes. The p-value is less than 0.001 under a 5% confidence level.

### Measurement performance

#### Pause rate prediction

We establish the dataset for XGBoost by using seven statistical features, as shown in Table 4. In the feature vector dataset, each vector contains seven dimensions of attributes, and the label *y* is the corresponding pause rate.

**Table 4:** Samples of the feature vector.

*v*	*γ*_1_	*γ*_2_	*θ*_1_	*θ*_2_	*θ*_3_	*θ*_4_	*y*
130	0	1	0.615384615	0.369230769	0.007692308	0.007692308	0.038461538
209	30	0	0.602870813	0.392344498	0.004784689	0	0.086124402
138	24	0	0.376811594	0.608695652	0.014492754	0	0
358	42	1	0.737430168	0.24301676	0.016759777	0.002793296	0.089385475
440	58	1	0.731818182	0.247727273	0.018181818	0.002272727	0.115909091

**Fig 9:**
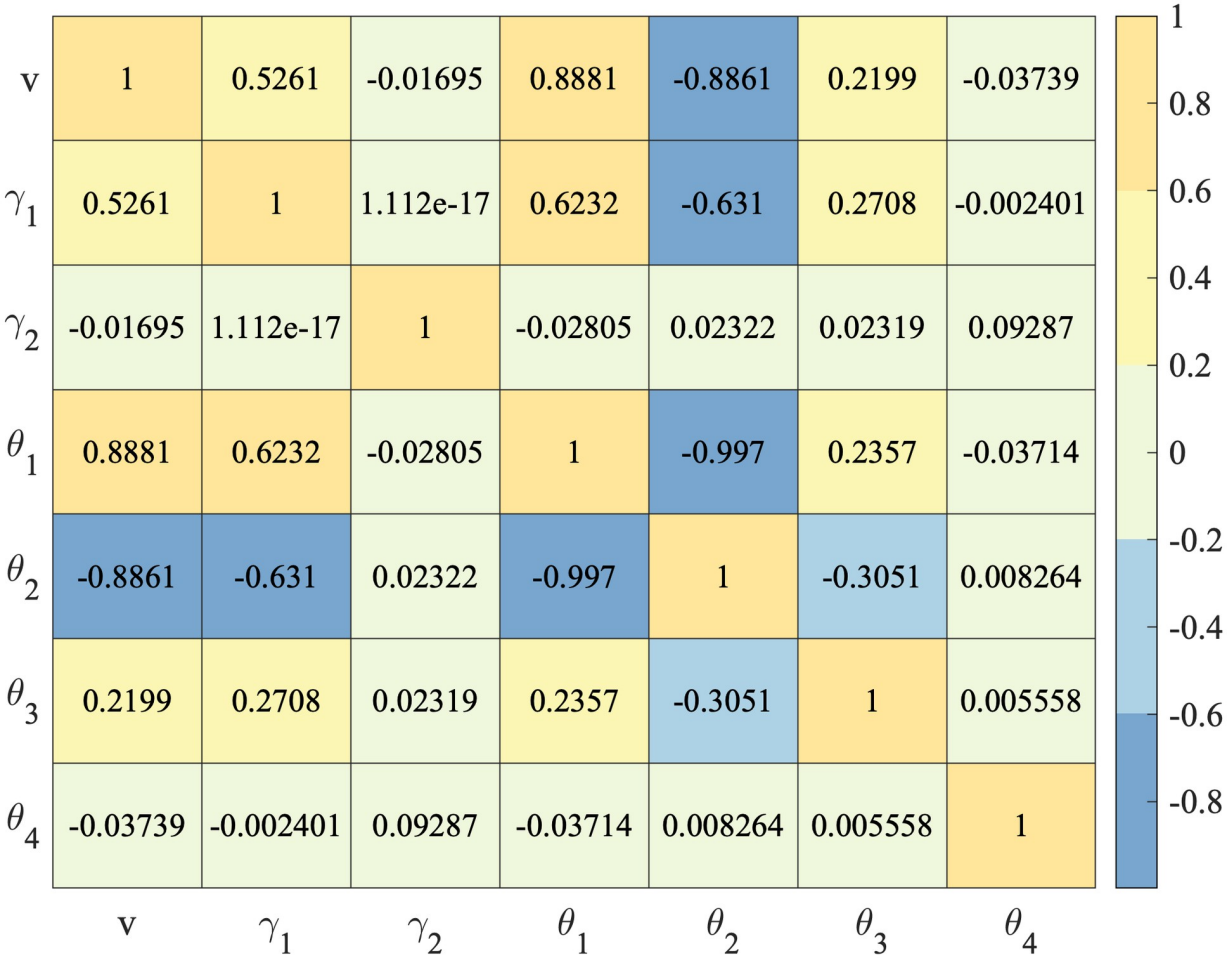
Correlation analysis of feature vector.

Before training the XGBoost model, we use Pearson correlation coefficient analysis to explore the correlation of these features and plot the heatmap, as shown in Fig 9. The color of the license plate feature *θ*, except *θ*_4_, has a positive and negative correlation with other features. In specific, the blue license plate rate *θ*_1_ has a positive correlation with time features and traffic flow, and the yellow license plate rate *θ*_2_ has a negative correlation with time features and traffic flow. In addition, the workday feature *γ*_2_ and the other color of the license plate rate *θ*_4_ have a very low correlation with other features.

The XGBoost regression algorithm has numerous parameters: the number of decision trees(n_estimators), learning rate(learn_rate), maximum depth of the tree(max_depth), minimum weight in leaf nodes(min_child_weight), the parameter that controls the number of leaves(gamma), the proportion of sample sampling(subsample), the scale of feature sampling(colsample_bytree). To find the optimal parameters, we use the grid search method combined with the K-fold cross-validation method. In this study, we set *K* = 5 to tune the model parameters. The search range and the optimal value of parameters are shown in Table 5.

**Table 5:** Optimal combination of parameters.

Parameter	Search range	Optimal value
n_estimators	[600, 2000]	700
learn_rate	[0.01, 0.1]	0.01
max_depth	[4, 25]	5
min_child_weight	[1, 7]	6
gamma	[0, 0.5]	0
subsample	[0.5, 1]	0.5
colsample_bytree	[0.5, 1]	1

**Fig 10:**
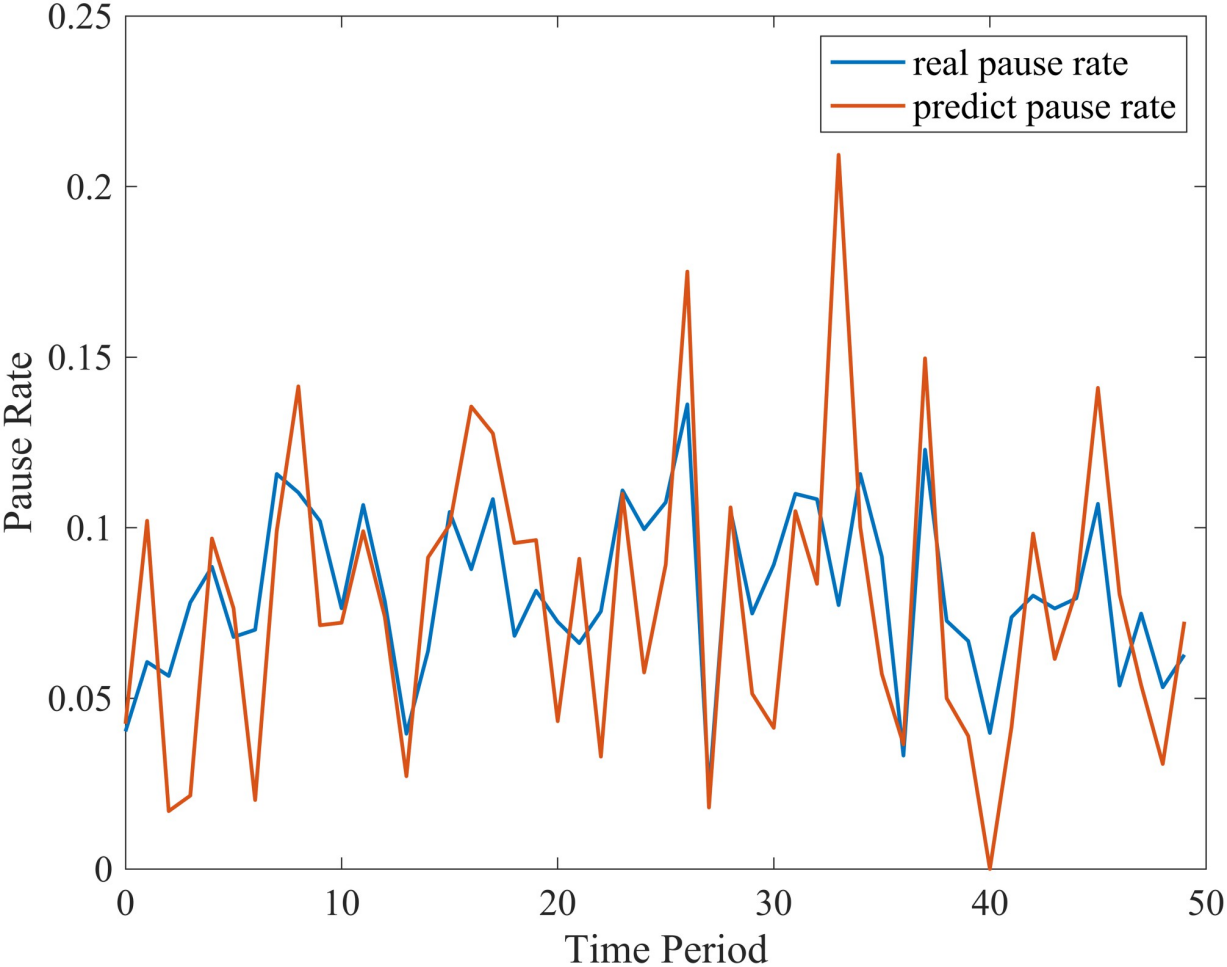
Prediction of pause rate.

To evaluate the effectiveness of the XGBoost model, we use RMSE and MAE to measure the prediction. The RMSE for the XGBoost model is 0.033, and the MAE is 0.026. As shown in Fig 10, the x-axis is time (slice a day into 96 periods), and the y-axis is pause rate. The blue line is the real pause rate, and the red is the prediction pause rate. Generally, the model has learned the pause rate changing characteristics well.

#### The bias regression

**Fig 11:**
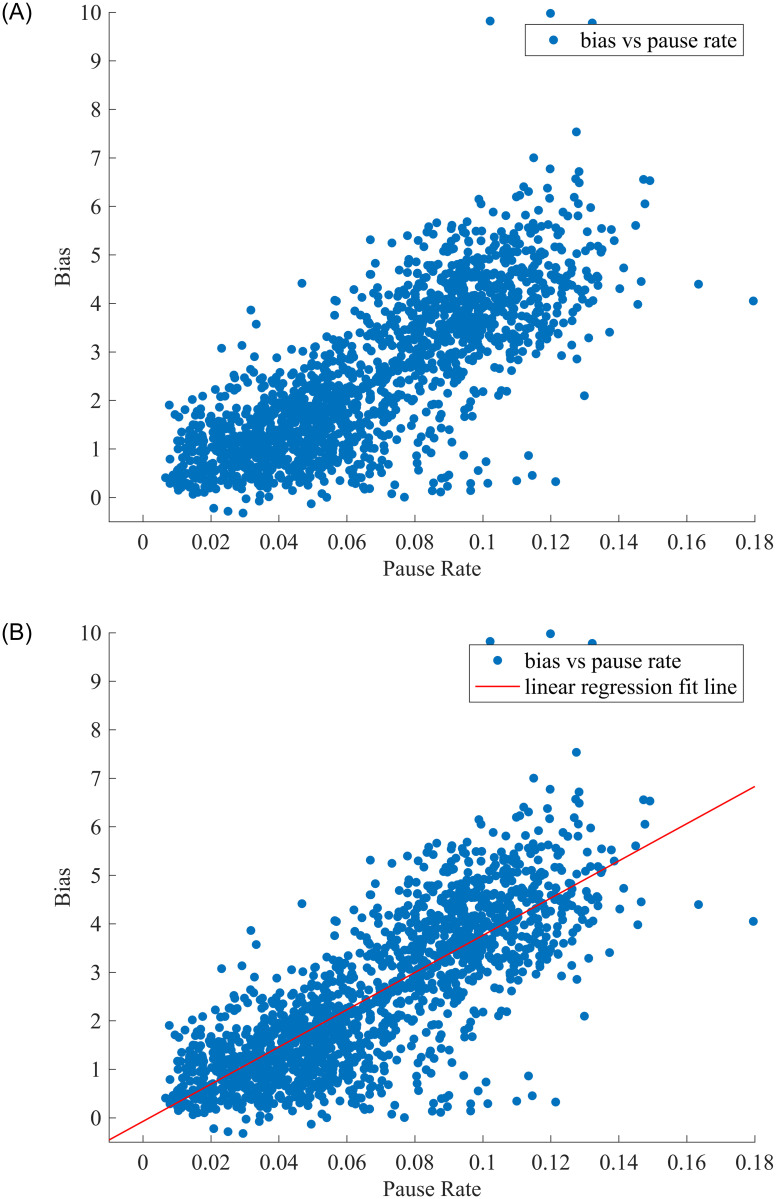
Scatterplot of bias versus pause rate and the regression line. A: Scatterplot of bias versus pause rate. B: Regression line.

In this work, we extract the pause rate from ETC transaction data and service area data by counting the number of vehicles per 15 minutes and plotting the scatter of the data, as shown in Fig 11.

In Fig 11(A), the x-axis is the pause rate, and the y-axis is biased. It is obvious that there is a linear trend; the bias is affected by the pause rate, and when the pause rate gets higher, the bias also gets higher. We use linear regression to fit the curve. The regression line is shown in Fig 11(B). The equation of the straight line is *y* = −0.07264 + 28.26*x*; the estimate of intercept is -0.07264, and the estimate of the slope is 38.36. The evolution metrics for linear regression are shown in Table 6.

**Table 6:** Evolution metrics for linear regression.

Multiple R	R Square	RMSE	SSE
0.81544612	0.66495237	0.9405	1489

#### The performance of correction term

**Fig 12:**
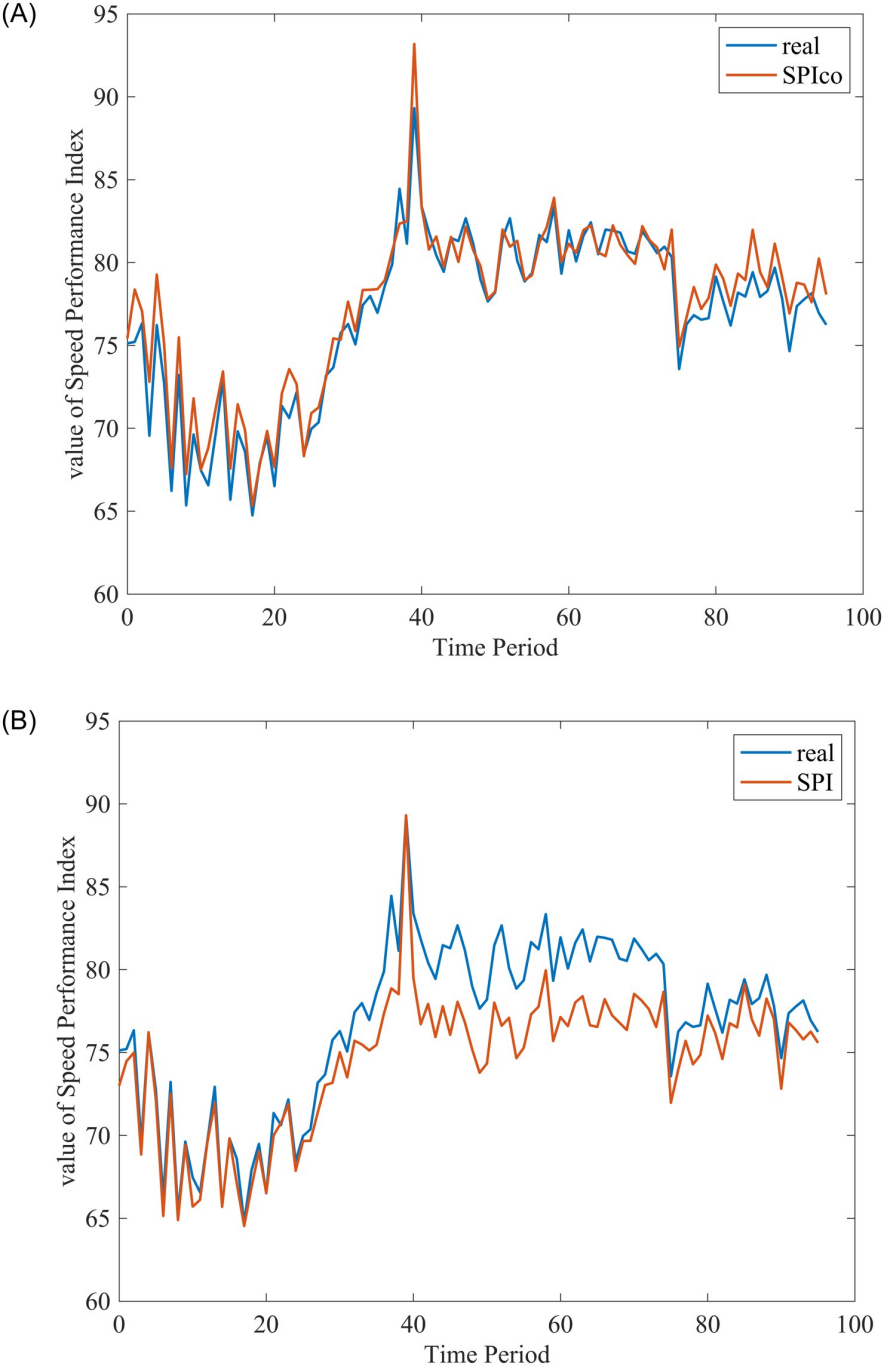
Comparison before and after adding the correction term. A: Comparison before adding the correction term. B: Comparison after adding the correction term.

After exploring the relevance between pause rate and bias and predicting pause rate, we add a correction term into SPI. To verify the new method, we choose data from day 13, May 2021, as shown in Fig 12, the red line is the raw traffic state, and in Fig 12(A), the blue line is the value of SPIco, in Fig 12(B), the blue line is the value of SPI. It is obvious that SPICo is more accurate under the situation of the service area segment.

Hence, we use the RMSE and MAE to evaluate the performance of the correction term. As shown in Table 7. The performance is better than the SPI under specific scenarios.

**Table 7:** Comparison of traffic congestion measurement.

Method	RMSE	MAE
SPI	2.87	2.36
SPIco	1.31	10.4

## Discussion and conclusion

In this work, we explore the influence of service areas on traffic congestion measurement. Then, we propose a comprehensive framework SPIco to improve the performance of traffic congestion measurement for the expressway. In the framework, we employ the XGBoost model to predict the pause rate; and then, we explore the relationship between bias and pause rate by linear regression. And at last, we propose a correction term into SPI. The main findings are as follows:

- The service area significantly influences the performance of traffic congestion measurement for expressways;
- The distribution of the travel time for users who don’t get into the service area is bimodal; the phenomenon is caused by a period of a day, like a free flow period and peak hour period;
- The proposed SPIco could be used to evaluate the expressway traffic states under the effect of service areas.

The proposed framework, SPIco, could be employed to measure expressway traffic congestion under the effect of service areas, which can also be applied into other traffic index measurement under the effect. However, there are still some limitations in this work: firstly, the value of R Square for linear regression is around 0.66, which is acceptable to some extent. However, the relationship between bias and pause rate may be more complex; secondly, we ignore the impact of weather and other sudden events. In the future work, we will do a more in-depth analysis to explore the relationship between bias and pause rate, making the pause rate prediction and traffic index measurement more accurately.
